# Reactivating nuclear PTEN to treat CLL

**DOI:** 10.18632/oncotarget.17543

**Published:** 2017-05-01

**Authors:** Rosa Bernardi, Paolo Ghia

**Affiliations:** Division of Experimental Oncology, San Raffaele Scientific Institute, Vita-Salute San Raffaele University, Milan, Italy

**Keywords:** chronic lymohocytic leukemia, TP53, PTEN, USP7, small molecule inhibitor

Chronic lymphocytic leukemia (CLL) is an indolent disease, though in a sizeable fraction of patients the outcome can be very poor with early and frequent need of therapy and the rapid development of resistance [4]. This is particularly true when aberrations (mutations or deletions) of the TP53 gene occur, correlating with a lack of response to immunochemotherapy. Recently, a number of novel non-chemotherapeutic agents have been approved for this subset of CLL patients, targeting relevant molecular pathways activated in the leukemic cells, such as the BcR pathway or the BCL2 antiapoptotic mechanism [4]. For these reasons, it is now strongly recommended to screen CLL patients in need of therapy for the presence of TP53 defects in order to avoid the use of chemotherapy in this unresponsive population (ww.ericll.org). Nevertheless, though responding very well to the novel inhibitors, relapsed/refractory patients carrying TP53 aberrations tend to have shorter time to progression and overall survival as compared to TP53-intact patients, thus revealing a still unmet clinical need. For this reason, the findings described in this issue by Carrà et al. [1] are of particular interest and may pave the way to more effective treatments for this subset of difficult-to-treat CLL patients. The authors focus their attention on the USP7-PTEN regulatory axis that appears to be constitutively active in CLL lymphocytes in the absence of specific gene defects. They find that overexpression of the deubiquitinase USP7 leads to PTEN inactivation in the leukemic cells and use of a small molecule targeting this axis induces cell cycle arrest and apoptosis of CLL cells independently of TP53 gene status.

USP7 (also known as HAUSP) is a deubiquitylase (DUB) that inhibits key tumor suppressive pathways and is overexpressed in different tumor types. Initially identified as a p53 DUB, it was later shown that, conversely, USP7 impairs p53 expression and function by regulating Mdm2 ubiquitylation [3], and in so doing exerts pro-oncogenic functions. In subsequent years, USP7 has been implicated in the regulation of other crucial tumor suppressors, not only by regulating protein availability, but also by modulating protein compartmentalization. This is the case of PTEN, which exerts important tumor suppressive functions both in the cytosol and in the nucleus, where it localizes in its monoubiquitylated form. USP7 was shown to act as a DUB of PTEN, thus forcing PTEN to the cytosol and hampering its tumor suppressive activities in the nucleus [6].

Different studies have recently reported that PTEN function is impaired in CLL, albeit not through gene mutation or promoter methylation [7]. Specifically, PTEN was found functionally inactivated through CK2-mediated phosphorylation [5], and downregulated through high expression of PTEN-targeting miRNAs [7]. Morotti and collaborators now describe a new mechanism leading to impaired PTEN function in CLL, which is caused by USP7 upregulation and impacts mainly on the nuclear activities of PTEN [1].

Carrà and coworkers identify several mechanisms that lead to increased USP7 expression and activation in CLL: USP7 is transcriptionally upregulated through miR-338-3p and miR-181b downregulation, and post-translationally stabilized through CK2-mediated phosphorylation, thus suggesting that increased USP7 expression might be of particular importance to CLL pathogenesis. Indeed, a small molecule inhibitor of USP7 recently tested in multiple myeloma (MM) pre-clinical models [2] blocks cell cycle progression and induces apoptosis also in CLL cells, similarly to specific USP7 downregulation [1]. Interestingly USP7 inhibition induces apoptosis in a p53-independent manner in CLL.

From a functional point of view, USP7 inhibition restores PTEN localization in CLL. PTEN localizes to both cytoplasm and nucleus of normal B lymphocytes, but is largely excluded from the nucleus of CLL B lymphocytes, and USP7 inhibition is able to restore the nuclear PTEN pool and to induce apoptosis in CLL B lymphocytes, while sparing normal B cells. Like USP7 inhibition, forcing PTEN to the nucleus is sufficient to induce apoptosis in CLL cells, thus strongly suggesting that apoptotic cell death caused by USP7 inhibition is mediated by reactivation of the nuclear pro-apoptotic function of PTEN [6], and that PTEN exerts tumor suppressive functions mostly from the nucleus in CLL.

In summary, the work described in this paper identifies USP7 as an important inhibitor of PTEN nuclear function in CLL and raises a number of interesting questions that may help to design with increased precision the potential use of USP7-inhibiting strategies for CLL. First and foremost, the findings herein described suggest that inhibiting USP7 may work irrespective of different PTEN levels in CLL, as lower PTEN expression has been described in patients with p53 aberrations. This point is of particular clinical relevance if one considers that low PTEN expression correlates with markers of worse prognosis and inferior time-to-treatment in CLL [8]. At the same time, evaluating PTEN levels, or p53 mutational status, may help to stratify patients for improved response to USP7 inhibition. Finally, as USP7 deregulation is mediated by several mechanisms in CLL, it would be important to understand whether CK2 inhibition mimics USP7 blockade, or may act cooperatively. All in all, further assessment of USP7 targeting interventions may hold new promise for CLL treatment alone or in combination with other novel inhibitors targeting concomitantly other pathways.
